# The influence of self-compassion on the mental health and well-being of women experiencing infertility: a systematic review

**DOI:** 10.1080/28324765.2026.2711571

**Published:** 2026-08-04

**Authors:** Shwikar Othman, Mitra Javanmard, Lauren Roberts, Lisa Conlon, Mary Steen

**Affiliations:** a School of Nursing and Midwifery, Edith Cowan University, Perth, Western Australia; b School of Medicine, College of Health, Adelaide University, Adelaide, South Australia; c Women and Newborn Health Service, King Edward Memorial Hospital, Western Australia; d Adelaide Nursing School, Faculty of Health and Medical Sciences, Adelaide University; e School of Nursing and Midwifery, College of Health, Adelaide University, Adelaide, South Australia

**Keywords:** Infertility, self-compassion, systematic review, mental health, well-being

## Abstract

Infertility can cause significant distress to women, increasing the risk of anxiety and depression. This systematic review examined the influence of self-compassion interventions on mental health and well-being among women experiencing infertility. The review was conducted in accordance with Joanna Briggs Institute (JBI) methodology and reported following PRISMA guideline. Databases searched included Medline, Embase, Ovid nursing database, Emcare, PsycINFO and Cochrane Library from inception to November 2024. Quality assessment was conducted using JBI critical appraisal tools. Title and abstract screening was conducted by three reviewers, and data extraction was performed independently by two reviewers. Sixteen studies met the inclusion criteria, seven intervention studies (*n* = 591 participants) and nine cross-sectional studies (*n* = 3005 participants). Intervention studies reported a reduction in depression, anxiety, shame and feelings of defeat, alongside improvements in self-compassion, well-being, quality of life (QoL), self-efficacy, emotion regulation difficulties and coping strategies. Meta-analysis was not undertaken due to substantial clinical, methodological and outcome heterogeneity across included studies. Cross-sectional studies indicated that higher self-compassion was associated with lower fertility-related stress and better psychological outcomes. Overall, the findings suggest that self-compassion interventions, including education-based approaches, may improve psychological health and resilience in women experiencing infertility. Incorporating multi-faceted education-based strategies to enhance self-compassion may provide further support and resilience for this population.

## Introduction

Self-compassion, a concept rooted in Buddhist philosophy for over 2500 years, has more recently gained recognition in Western societies for its role in promoting health and well-being (Neff, 2003). Self-compassion involves treating oneself with kindness, recognising shared human experiences and maintain mindful awareness during times of difficulty (Neff, 2003, p. 87). Theoretically, self-compassion is understood as an adaptive emotion regulation strategy that buffers against negative self-evaluation and psychological distress. It has been consistently associated with lower levels of anxiety, stress and depression, alongside improvements in self-work, happiness and interpersonal relationships (Booth et al., 2019; Ferrari et al., 2018; Neff, 2003).

Infertility is a significant global health concerns, affecting approximately 48.5 million couples worldwide (World Health Organization, 2023a). In Australia, one in six couples experience infertility, with prevalence increasing due to a range of social and medical factors (IVF Australia, 2023), Genea Fertility SA (Genea Fertility SA, 2018). The World Health Organisation defines infertility as ‘the failure to achieve a pregnancy after 12 months or more of regular unprotected sexual intercourse’ (World Health Organization, 2023b). Beyond its medical definition, infertility is widely recognised as a psychological challenging life event (Chen et al., 2004; Chernoff et al., 2021; Cousineau & Domar, 2007; Kang et al., 2022; Rooney & Domar, 2018; Sormunen et al., 2018). Women, in particular, experience elevated rates of mental health disorders, including generalised anxiety disorder (23.2%), major depression (17.0%) and dysthymia (9.8%) (Chen et al., 2004). Infertility has been conceptualised as a developmental crisis, disrupting life expectations and identity (Cousineau & Domar, 2007; Sexton et al., 2010). Although both partners are affected, women often report greater emotional burden, including heightened stress and social pressure (Ferreira et al., 2015; Galhardo et al., 2013b).

Many individuals pursue assisted reproductive technologies such as in vitro fertilisation (IVF) which introduces additional physical, emotional and financial demands (Bakhtiyar et al., 2019; Kaliarnta et al., 2011). These challenges highlight the importance of psychological interventions that support coping and emotional resilience. Approaches targeting emotion regulation, mindfulness and adaptive coping, including self-compassion, have shown promise in improving psychological outcomes and quality of life during fertility treatment (Berking et al., 2008; Haica, 2013).

From a theoretical perspective, self-compassion may be relevant in infertility, as it targets maladaptive processes such as self-criticism, shame and rumination, which are commonly reported in this population (Cousineau & Domar, 2007; Galhardo et al., 2013b). It aligns with established stress and coping frameworks, functioning as a protective factor that supports emotional resilience and adaptive coping responses (Berking et al., 2008; Neff & Germer, 2013). Evidence from broader populations demonstrates that higher self-compassion is associated with reduced psychological distress and improved emotional regulation (Delaney, 2018; Ferrari et al., 2018), suggesting potential applicability within infertility contexts.

The current evidence base includes both intervention and observational studies. Cross-sectional studies provide insights into the associations between self-compassion and psychological outcomes, including stress, anxiety and quality of life (Ferreira et al., 2015; Galhardo et al., 2013b), while intervention studies evaluate the effectiveness of strategies designed to enhance self-compassion and coping (Berking et al., 2008). Considering both forms of evidence allows for a more comprehensive understanding of the potential role of self-compassion in supporting women experiencing infertility.

To date, no systematic review has specifically examined the effectiveness of self-compassion interventions for women experiencing infertility and/or undergoing IVF treatment. Therefore, this review aimed to evaluate current evidence on self-compassion interventions, including education and training programme, for improving psychological outcomes in this population. A secondary aim was to assess the role of educational approaches in developing self-compassion skills. The findings will inform the development of future interventions, including a targeted self-compassion intervention for women experiencing infertility.

Review questions:

1. What evidence exists on the effectiveness of self-compassion interventions on the mental health and well-being of women experiencing infertility?
2. What is the influence of providing self-compassion education and training as an intervention on the mental health and well-being of women experiencing infertility?

## Review methods

This systematic review was conducted in accordance with the Joanna Briggs Institute (JBI) methodology and reported following the Preferred Reporting Items for Systematic Reviews and Meta-Analyses (PRISMA) guidelines (Page et al., 2021). The protocol for this review was published with PROSPERO (ID: CRD42022330158) (Javanmard et al., 2022).

### Inclusion criteria

#### Types of included studies

The current review included studies that assessed the influence of self-compassion interventions and/or measurement of self-compassion levels in women experiencing infertility. Eligible study designs included randomised controlled trials, quasi-experimental studies, cohort studies, case-controlled studies, pre- and post-studies, mixed-method studies and cross-sectional studies. Cross-sectional studies were included to examine associations between self-compassion and psychological outcomes. Studies were restricted to those published in English, from inception to November 2024.

#### Types of participants

Studies involving women experiencing primary or secondary infertility were included. Primary infertility refers to difficulty conceiving in someone who has never conceived, while secondary infertility refers to difficulty conceiving one or more previous pregnancies (National Health Service (NHS), 2023).

#### Types of interventions

Studies were included if they involved interventions aimed at measuring and/or enhancing self-compassion through education or training targeted at women experiencing infertility (e.g. programmes, workshops or sessions). The interventions could be delivered independently or alongside other approaches and included face-to-face, one-to-one, group-based or digital formats. Interventions were facilitated by health professionals or trained educators including health workers, social workers, counsellors, psychologists, nurses, midwives, meditation practitioners or mindfulness trainers.

#### Outcomes measures

Eligible studies included those assessing self-compassion (e.g. using validated scales), its subcomponents (self-kindness, common humanity, mindfulness), fear of compassion and psychological outcomes such as anxiety, depression, stress, quality of life or overall wellbeing. Studies evaluating self-compassion interventions or training programmes were also included.

### Exclusion criteria

Systematic reviews, scoping reviews, study protocols, conference abstracts without full text and qualitative studies were excluded. Qualitative studies were excluded to maintain methodological consistency and focus on quantifiable outcomes, enabling comparison across studies and supporting structured synthesis of intervention effectiveness and associations.

### Information sources and search strategy

A comprehensive systematic search of both published and unpublished studies was undertaken across six electronic databases (Medline, Embase, Ovid nursing database, Emcare, PsycINFO and the Cochrane Library) from inception to November 2024, (See Appendix A: Medline database search strategy). The search strategy combined controlled vocabulary (e.g. MeSH terms) and free-text keywords related to infertility and self-compassion. Infertility terms included Infertility, Assisted Reproductive Techniques, Fertilisation in Vitro, Embryo Transfer, Intracytoplasmic Sperm Injection and Artificial Insemination, alongside keywords such as infertil *, subfertil *, IVF, ICSI and assisted reproduct *. Self-compassion terms included Self-Compassion and keywords such as self-compassion, self-kindness, self-worth, self-acceptance, self-care and self-forgiveness.

In addition, grey literature was searched using Google Scholar was, with the first ten pages (*n* = 100) screened to identify any relevant studies.

### Selection process

**Figure 1.**
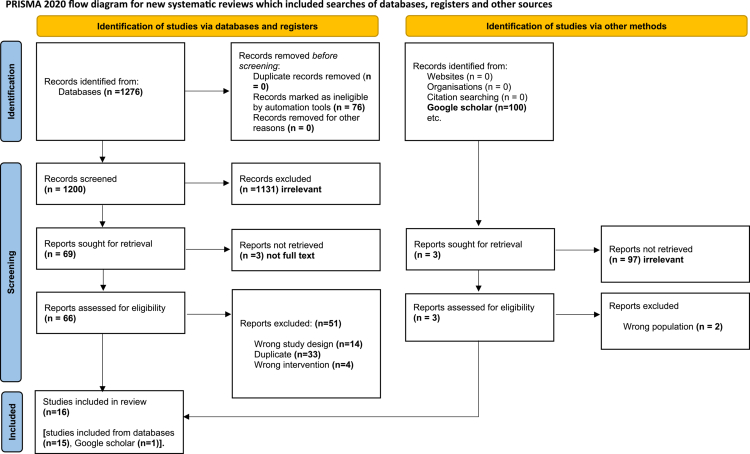
PRISMA flowchart of the systematic review process.

All search results were imported into EndNote citation management software (The Endnote Team, 2013), and then uploaded to Covidence (Veritas Health Innovation, 2023), where duplicates were removed electronically. Four review authors (SO, MJ, LR, MS) independently screened titles and abstracts. Full-text articles were then assessed independently against the eligibility criteria. Disagreements between reviewers were resolved through discussion and consensus, with any discrepancies discussed until mutual agreement was reached. Inter-reviewer agreement was assessed throughout the screening process to ensure consistency and reliability in the study selection. While a formal inter-rater agreement statistic was not computed, as disagreements were few and a consensus-based approach was used (Figure 1: PRISMA flowchart).

### Data extraction

Data extraction was conducted independently by two review authors (MJ, LR) using a customised Excel spreadsheet. A pilot test of two randomly selected studies was undertaken to refine the extraction process. Data extracted included authors, title, year of publication and country of origin; study characteristics: study aims, study design and methods, study inclusion and exclusion, recruitment strategy; participant details: sample size, scales used, key findings, study conclusions.

For the intervention studies, additional details were extracted, including intervention characteristics, delivery mode, comparator if reported, pre- and post-measurement findings, reported outcomes and the effectiveness of the intervention. Extracted data were checked by a third reviewer (SO) for accuracy and completeness, and any discrepancies were discussed to reach a consensus with an author (MS).

### Assessment of methodological quality and critical appraisal

All final included studies were assessed and critically appraised using the Joanna Briggs Institute critical appraisal tools appropriate to study design (Tufanaru et al., 2020). These included checklists for randomised controlled trials (13 items), quasi-experimental studies (9 items) and cross-sectional studies (8 items). The JBI checklist for randomised controlled trials assesses methodological quality, including randomisation, allocation concealment, blinding follow up and outcome measurement and statistical analysis. Each item was rated as ‘yes’, ‘no’, ‘unclear’, or ‘not applicable’.

Overall, intervention studies were assessed as high quality, meeting at least 85% of the appraisal criteria, indicating low risk of bias. One study was rated as moderate quality, meeting approximately 61% of criteria, due to blinding and study design Galhardo et al. (2013a). Cross-sectional studies were also generally rated as high quality; however, common methodological limitations were noted, including reliance on self-reported measures and potential selection bias (Appendix B: Critical Appraisal of the included studies). All included studies were also categorised according to their level of evidence based on study design using JBI level of evidence (Aromataris et al., 2024; Tufanaru et al., 2020).

## Results

### Study selection

A total of 1276 studies were identified across six databases. After removing 76 duplicates, 1200 studies were screened by title and abstract, with 69 studies deemed eligible for full-text screening. Of these 54 studies were excluded for the following reasons: duplicate records not identified during the initial screening process (*n* = 33), wrong study design (*n* = 14), wrong intervention (*n* = 4) and unavailable full text (*n* = 3). Only 15 studies from the database search met the inclusion criteria.

Searching Google Scholar identified 100 studies, of which only three were retrieved for full-text screening. Two of these studies were excluded, leaving only one study to be included in this review. Overall, 16 studies met the inclusion criteria and were included in this review (Figure 1 PRISMA flowchart).

Meta-analysis was not undertaken due to substantial clinical, methodological and outcome heterogeneity across studies, which would render any pooled estimate difficult to interpret and potentially misleading. Instead, a narrative synthesis was conducted to preserve important contextual differences between studies.

### Study characteristics

Sixteen studies were included in this review, seven intervention studies (Afshani et al., 2019; Galhardo et al., 2013a; Li et al., 2016; Njogu et al., 2023, Rezapour-Mirsaleh et al., 2020, 2024; Sahraian et al., 2024) and nine cross-sectional studies (Galhardo et al., 2011, 2013b; Hajihasani & Ekhtiari Amiri, 2023; Hoyle et al., 2022; Pinto-Gouveia et al., 2012; Raque-Bogdan & Hoffman, 2015; Sadiq et al., 2022; Schuette et al., 2023; Sotoudeh et al., 2022). Among the intervention studies, three were randomised controlled clinical trials (Njogu et al., 2023; Rezapour-Mirsaleh et al., 2024; Sahraian et al., 2024), one was a controlled clinical trial with a pre- and post-design (Galhardo et al., 2013a) and three were quasi-experimental studies. Overall, there was a considerable heterogeneity in study designs and methods (Afshani et al., 2019; Li et al., 2016; Rezapour-Mirsaleh et al., 2020).

The included studies were conducted across several countries, with six undertaken in Iran (Afshani et al., 2019; Hajihasani & Ekhtiari Amiri, 2023, Rezapour-Mirsaleh et al., 2020, 2024; Sahraian et al., 2024; Sotoudeh et al., 2022), four studies in Portugal (Galhardo et al., 2011, 2013a, 2013b; Pinto-Gouveia et al., 2012), three in the United States (Hoyle et al., 2022; Raque-Bogdan & Hoffman, 2015; Schuette et al., 2023), one each in China, Kenya and Pakistan (Li et al., 2016; Njogu et al., 2023; Sadiq et al., 2022). All included studies were published between 2011 and 2024. All intervention studies were conducted across diverse settings including a university research unit, reproductive sciences institute, fertility medical centre within hospital obstetrics and gynaecology outpatient clinics (Afshani et al., 2019; Galhardo et al., 2013a; Li et al., 2016; Njogu et al., 2023; Rezapour-Mirsaleh et al., 2020, 2024; Sahraian et al., 2024). The cross-sectional studies were conducted in infertility centres or in public and private clinics (Galhardo et al., 2011, 2013b; Hajihasani & Ekhtiari Amiri, 2023; Pinto-Gouveia et al., 2012; Sadiq et al., 2022; Schuette et al., 2023; Sotoudeh et al., 2022), while two studies were based on global online surveys (Hoyle et al., 2022; Raque-Bogdan & Hoffman, 2015) (Tables 1 and 2 Study characteristics).

**Table 1.** Characteristics of interventional studies (*n* = 7).

Author (year) country	Aim of study	Study design (level of evidence)	Eligibility criteria and setting	Sample and group characteristics	Control group	Timepoints and measures (scales)	Intervention(s)	Key findings
Rezapour-Mirsaleh et al. (2024) Iran	To compare the effects of self-compassion interventions based on religious and non-religious perspectives on anxiety and quality of life in infertile women	Randomised controlled trial (three arms: religious SC, non-religious SC, control); Level of evidence (1)	Inclusion criteria: 25–40 years, ≥5 years infertility, ≥3 years treatment, Islamic orientation, minimum education. Exclusion criteria: mental/personality disorders, psychiatric medication, other psychological treatment. Setting: Yazd Reproductive Sciences Institute	Total *N* = 78; women with primary infertility undergoing IVF Intervention groups: Religious self-compassion (*n* = 26) Non-religious self-compassion (*n* = 26)	Control group (*n* = 26); wait-list and usual care.	T1 (pre), T2 (post), T3 (2-month follow-up). Measures: no SC scale QOLICQ BAI	Eight sessions of self-compassion training (religious vs non-religious formats), focusing on mindfulness, self-kindness, common humanity, emotional regulation and acceptance; religious group integrated Islamic teachings.	Interventions (religious and non-religious) significantly improved outcomes compared with control: QoL: significant time × group effect (F = 75.58, *p* < 0.001) Anxiety: significant time × group effect (F = 23.08, *p* < 0.001) Mean changes (T2-T1) QoL: + 30.65 (religious SC), + 16.92 (non-religious SC), −1.92 (control) Anxiety: −5.69 (religious SC), −3.92 (non-religious SC), −0.42 (control) Follow-up changes (T3-T1): QoL: + 32.65 (religious), + 19.04 (non-religious), −1.50 (control) Anxiety: −6.15 (religious), −3.73 (non-religious), −0.15 (control) Religious self-compassion produced significantly greater improvements than non-religious self-compassion (QoL *Δ* = 13.73; anxiety *Δ* = −1.77 at post-test, *p* < 0.01)
Sahraiain et al. (2024) Iran	To explore whether Mindful Self-Compassion (MSC) therapy reduces psychological distress and mental health symptoms in women undergoing IVF	Two-arm double-blind randomised controlled trial; Level of evidence (1)	Inclusion: ≥diploma education, ≥1 year infertility diagnosis, no chronic illness or psychiatric disorder. Exclusion: missing ≥2 sessions, illness, other treatments, or withdrawal. Setting: Shiraz Infertility Centre	Total *N* = 57; all women undergoing IVF Intervention group: MSC (*n* = 29; completed *n* = 23); mean age 33.36 (range 18–45)	Control group (*n* = 28; completed *n* = 22); usual care including IVF treatment and routine psychosocial support; mean age 34.72.	T1 (pre), T2 (post), T3 (2-month follow-up). Measures: No SC scale SCL-90-R Snyder's Hope Scale Satisfaction scales (Likert)	Eight weekly MSC sessions (2 h/session) plus half-day retreat (4 h); focused on mindfulness, self-compassion, emotional regulation, relationships and loving-kindness practices.	Interventions significantly improved psychological outcomes compared with control, with effects maintained at 2-month follow-up: hope (hopelessness): T2: t = 10.06, *p* < 0.001, d = 0.85 T3: t = 12.21, *p* < 0.001, d = 0.87 Anxiety: T2: t = 5.48, *p* < 0.001, d = 0.63 T3: t = 6.46, *p* < 0.001, d = 0.69 Depression: T2: t = 5.21, *p* < 0.001, d = 0.61 T3: t = 3.88, *p* < 0.001, d = 0.50 Anger-hostility: T2: t = 6.34, *p* < 0.001, d = 0.68 T3: t = 4.47, *p* < 0.001, d = 0.55 Interpersonal sensitivity: T2: t = 3.05, *p* < 0.001, d = 0.41 T3: t = 6.70, *p* < 0.001, d = 0.70 Clinical improvement rates (MSC): T2: 88% (hope), 64% (anger), 68% (anxiety), 68% (depression), 84% (interpersonal sensitivity) T3: 88%, 56%, 64%, 84% and 72%, respectively Participant feedback: 74.4% satisfied 65.6% would recommend MSC
Njogu et al. (2023) Kenya	To examine the effects of self-compassion training using videos (SCV) versus self-compassion training using digital stories (SC-DS) as compared to a control group (CG) on reducing anxiety and depression symptoms among women pursuing fertility treatment.	Multi-centre, three-arm randomised controlled trial; Level of evidence (1)	Inclusion criteria: Women aged 22–44, diagnosed with infertility, receiving treatment, able to read Kiswahili, follow instructions and access the internet. Setting: three Kenyan hospitals	Total *N* = 200 SCV (*n* = 65) SC–DS (*n* = 67) Participants: primary infertility (23%–25%) secondary infertility (75%–77%) majority infertility duration 1–6 years (~80%)	Control group (*n* = 68); usual care Primary infertility 23.53%; secondary infertility 76.47%; infertility duration 1–6 years 82.36%	T1 (baseline), T2 (post-intervention), T3 (10-week follow-up) Measures: SCS–SF Zung Anxiety Scale Zung Depression Scale Infertility Self-Efficacy Pregnancy outcomes	8-week online self-compassion programmes: SCV: two daily 5-minute videos (guided meditation + psychoeducation) SC–DS: eight 1-hour sessions using digital storytelling Both targeted mindfulness, self-kindness, emotional regulation, cognitive restructuring and daily practice (10-45 minutes).	Interventions (SCV and SC–DS) significantly improved psychological outcomes compared with control: anxiety and depression: reduced from T1–T2 and T1–T3 (*p* < 0.001; d > 0.8) Self-compassion: increased (SC-DS vs control: MD = 21.32; 95% CI 18.64–24.58; d = 2.192) Between-group effects (SC-DS vs control): Anxiety: T2: MD = −8.38 (95% CI −9.46 to −7.30; d = 2.679) T3: MD = −11.63 (−13.13 to −10.14; d = 2.754) Depression: T2: MD = −17.66 (−19.64 to −15.68; d = 3.013)
Rezapour-Mirsaleh et al. (2020) Iran	To determine the effect of self-compassion intervention based on religious perspective on the anxiety and quality of life of infertile women.	Quasi-experimental pre-test/post-test design with experimental and control groups; Level of evidence (2)	Inclusion: ≥5 years infertility, ≥3 years treatment, age 25–40, Islamic orientation, minimum school education. Exclusion: mental/personality disorders, psychiatric medication, or other psychological treatment. Settings: Yazd reproductive sciences institute.	Total *N* = 24 IG (*n* = 12); women with primary infertility.	Control group (*n* = 12); wait-list receiving usual medical care and minimal informational support.	T1 (pre), T2 (post). Measures: No SC scale. QOLICQ BAI	Eight face-to-face sessions of self-compassion intervention based on Islamic teachings, focusing on self-kindness, mindfulness, acceptance, emotional regulation, empathy, self-forgiveness and meaning-making, with homework exercises.	Intervention significantly improved outcomes compared with control: Anxiety: decreased (F = 68.54, *p* < 0.001, η^2^ = 0.77) Overall quality of life: increased (F = 57.22, *p* < 0.001, η^2^ = 0.74) Quality of life subdomains: Physical: F = 20.01, *p* = 0.001, η^2^ = 0.57 Emotional: F = 6.66, *p* = 0.002, η^2^ = 0.31 Social: F = 14.43, *p* = 0.02, η^2^ = 0.49 Mental: F = 32.45, *p* = 0.001, η^2^ = 0.68 Sexual: F = 7.04, *p* = 0.01, η^2^ = 0.32 Spiritual/religious: F = 6.01, *p* = 0.02, η^2^ = 0.28
Afshani et al. (2019) Iran	To investigate the effect of teaching self-compassion on the psychological well-being of initial infertile women.	Quasi-experimental pre-test/post-test design with control group; Level of evidence (2)	Inclusion: primary infertility; age < 35 years. Exclusion: > 2 session absences; failure to complete homework (>3 times). Settings: Yazd Reproductive Sciences Institute	Total *N* = 32 IG (*n* = 16) Mean age not reported.	Control group (*n* = 16); usual care.	T1 (pre), T2 (post). Measure: No SC scale reported. Ryff's Psychological Well‑Being Questionnaire	Eight face-to-face sessions (90 min each) focusing on self-compassion components (mindfulness, self-kindness, common humanity), with emphasis on reducing self-criticism and improving emotional regulation.	Self-compassion training significantly improved psychological well-being compared with control: between-group (post-test): IG = 349.88 vs control = 320.88 (*p* = 0.04) Within intervention group: increased from 316.94 (T1) to 349.88 (T2) (*p* = 0.01) ANCOVA: significant group effect (*p* = 0.01); intervention accounted for 72.7% of variance (η^2^ = 0.727) Control group: no significant change (*p* = 0.15)
Li et al. (2016) China	To investigate the effectiveness of a mindfulness-based intervention (MBI) among women subjected to first IVF treatment at a fertility medical centre in China.	Quasi-experimental pre-test/post-test design with control group; Level of evidence (2)	Inclusion: first IVF treatment, willingness to participate, able to complete questionnaires, no prior mental health diagnosis/treatment, no prior yoga or meditation experience. Settings: Fertility medical centre in Southwest Hospital in Chongqing.	Total *N* = 108 IG (*n* = 58); mean age 30.66 ± 4.29; infertility duration 4.66 ± 3.06 years; treatment duration 2.34 ± 1.63 years.	Control group (*n* = 50); routine care; mean age 30.70 ± 4.77; infertility duration 5.20 ± 3.03 years; treatment duration 2.68 ± 1.53 years.	T1 (pre), T2 (post); pregnancy rates at 6-month follow-up. Measures: SCS FFMQ FertiQoL DERS COMPI coping scales	6 weekly face-to-face MBI sessions (2–2.5 h), including mindfulness meditation (body scan, breathing, yoga, walking), emotional awareness and coping skill development; supported with manual and home practice.	Intervention significantly improved psychological and clinical outcomes compared with control: Self-compassion: Increased from 3.06 to 3.60 (*p* < 0.001) Quality of life (FertiQoL): increased from 59.04 to 71.72 (*p* < 0.001) Emotion regulation difficulties: decreased from 92.00 to 78.76 (*p* < 0.001) Coping strategies: meaning-based coping ↑ (*p* < 0.001) Active avoidance ↓ (*p* = 0.001) Passive avoidance ↓ (*p* = 0.010) Active confronting: no change (*p* = 0.959) Pregnancy rate: 44.83% (MBI) vs 26.00% (control); *p* = 0.04 No significant changes in the control group
Galhardo et al. (2013a) Portugal	To evaluate the efficacy of the Mindfulness-Based Programme for Infertility (MBPI) among women undergoing infertility treatment	Controlled clinical trial with pre-/post-test design; Level of evidence (1).	Inclusion: ≥18 years; diagnosed infertility. Settings: A university research unit.	Total *N* = 92 IG group (*n* = 55); women with primary infertility; mean age 34.87 ± 4.20; marriage duration 6.62 ± 3.57 years; infertility duration 3.35 ± 2.53 years.	Control group (*n* = 37); mean age 33.14 ± 3.94; marriage duration 5.70 ± 3.25 years; infertility duration 3.05 ± 2.44 years.	T1 (pre), T2 (post; 10-week interval). Measures: SCS BDI STAI-Y1 ESS OAS Entrapment Scale Defeat Scale AAQ-II ISE FMI	MBPI: 10 weekly face-to-face sessions (2 h/session; one full-day session), including mindfulness practices, psychoeducation, values clarification and emotional regulation; partners attended 3 sessions.	Intervention significantly improved psychological outcomes compared with control: depression (BDI): 11.02 → 6.18 (*p* < 0.001) Anxiety (STAI): 47.82 → 43.02 (*p* = 0.009; no significant time × group interaction) Self-judgement: 38.49 → 34.38 (*p* < 0.001) Entrapment: external *p* = 0.009; internal *p* = 0.021 Defeat: *p* = 0.007 Experiential avoidance: *p* < 0.001 Internal shame: *p* = 0.001 External shame: *p* = 0.011 Infertility self-efficacy (ISE): 81.87 → 98.87 (*p* < 0.001) Mindfulness (subgroup): 34.00 → 38.40 (*p* < 0.001) Self-compassion: no significant change Control group: no significant improvements, except reduced self-judgement

**Intervention group (IG)**

**Control group (CG)**

List of abbreviated scales: QOLICQ – Quality of Life in Infertile Couples Questionnaire; BAI – Beck Anxiety Inventory; SCS – Self-Compassion Scale; FFMQ – Five Facet Mindfulness Questionnaire; FertiQoL – Fertility Quality of Life Questionnaire; DERS – Difficulties in Emotion Regulation Scale; COMPI Coping Scales – Copenhagen Multi-Centre Psychosocial Infertility Coping Strategy Scales; BDI – Beck Depression Inventory; STAI-Y1 – State–Trait Anxiety Inventory, Form Y-1; ESS – External Shame Scale; OAS – Other As Shamer Scale; Entrapment Scale – Entrapment Scale; Defeat Scale – Defeat Scale; AAQ-II – Acceptance and Action Questionnaire–II; ISE – Infertility Self-Efficacy Scale; FMI – Freiburg Mindfulness Inventory.

**Table 2.** Characteristics for the cross-sectional studies (*n* = 9).

Author (year) country	Aim of study	Study design (level of evidence)	Eligibility criteria and setting	Sample characteristics	Measures (scales)	Key findings
Hajihasani and Ekhtiari Amiri (2023) Iran	Examine the role of marital relationship quality and self-compassion in psychological distress in infertile women	Quantitative, cross-sectional descriptive-analytic level of evidence (4)	Inclusion: infertility ≥1 year; women aged 20–40 Exclusion: severe illness; history of mental illness Setting: fertility clinics, Iran	*N* = 400 infertile women Mean age: 28.88 ± 6.60 (20–40) 90% urban; 10% rural 63% ≥junior college Marriage duration: 5.80 ± 2.72 years Infertility duration: 3.00 ± 1.95 years	SCS DASS MRQ Demographics	Higher self-compassion associated with lower psychological distress (*p* < 0.01) Self-compassion predicted psychological distress (b = 0.08, *p* < 0.05) Marital relationship quality negatively associated with psychological distress (*p* < 0.01) Marital relationship quality predicted psychological distress (b = 0.49, *p* < 0.001) Model explained 29.9% variance in psychological distress
Schuette et al. (2023) USA	Examine associations between modifiable psychological variables and depression, anxiety and post-traumatic growth (PTG) in women experiencing infertility.	Quantitative, cross-sectional; path analysis (fully saturated SEM) Level of evidence (4)	Inclusion: assigned female at birth; self-identified infertility; ≥18 years; US resident Exclusion: not reported Setting: Duke Fertility Centre; online recruitment (social media, ResearchMatch, Qualtrics panels)	*N* = 457 Mean age: 32.30 ± 5.16 Duration trying to conceive: <1 year *n* = 41 1–5 years *n* = 291 >5 years *n* = 48 Children: *n* = 343 no; *n* = 113 yes Miscarriage: *n* = 216 yes; *n* = 239 no	SCS-SF PROMIS Depression PROMIS Anxiety PTGI AAQ-II IUS-SF mDES CSI-4 FFMQ-SF	Lower self-compassion predicted higher depression (b = −0.22, *β* = −0.13, *p* = 0.004) Higher self-compassion predicted higher PTG (b = 0.71, *β* = 0.35, *p* < 0.001) Mindfulness showed indirect effects on depression (*p* = 0.005) and PTG (*p* < 0.001) via self-compassion Models explained variance in depression (51.3%), anxiety (46.7%) and PTG (25.8%)
Hoyle et al. (2022) USA	Examine the association between adaptive self-management traits (including self-compassion) and psychological distress in women experiencing infertility	Quantitative, cross-sectional Level of evidence (4)	Inclusion: living in the US; currently experiencing infertility Exclusion: incomplete data or careless responding Setting: online infertility groups and forums	*N* = 326 women Mean age: 33.36 ± 4.35 Number of months trying to conceive (square-root transformed): M = 5.74, SD = 2.05 Number of unsuccessful treatment cycles: M = 1.91 ± 1.46	SCS-SF ERQ Positivity Scale CES-D-R K6	Higher self-compassion associated with lower depressive symptoms (r = −0.34 to −0.49, *p* < 0.01) Self-compassion was not a significant direct predictor of depressive symptoms (*p* = 0.717) Low self-coldness (self-compassion facet) predicted lower depressive symptoms (B = −0.20, *p* < 0.001) Self-compassion attenuated the association between primary infertility and depressive symptoms (interaction B = −0.20, *p* = 0.030) Lower expressive suppression buffered the effect of primary infertility on distress (B = −0.21, *p* = 0.002) Positivity negatively predicted depressive symptoms (B = −0.38, *p* < 0.001)
Sadiq et al. (2022) Pakistan	Examine the relationship between marital quality, self-compassion and psychological distress in women with primary infertility	Quantitative, cross-sectional correlational Level of evidence (4)	Inclusion: first wives; cohabiting; marriage ≥3 years; ≥1 year treatment; regular intercourse without contraception Exclusion: comorbid physical or psychological conditions Setting: public and private hospitals/clinics, Pakistan	*N* = 115 women with primary infertility Mean age: 29.08 ± 3.91 Mean infertility duration: 4.00 ± 3.46 years	SCS-SF RAS DASS Demographics	Higher self-compassion associated with lower psychological distress (r = −0.61, *p* < 0.01) Marital quality positively associated with self-compassion (r = 0.54, *p* < 0.01) and negatively associated with psychological distress (r = −0.49, *p* < 0.01) Self-compassion negatively predicted depression (b = −0.56, *p* < 0.001), anxiety (b = −0.22, *p* < 0.05) and stress (b = −0.38, *p* < 0.01) Self-compassion mediated the relationship between marital quality and psychological distress (depression and stress) Models explained variance in depression (R^2^ = 0.47), anxiety (R^2^ = 0.08) and stress (R^2^ = 0.24)
Sotoudeh et al. (2022) Iran	Examine relationship between psychological acceptance, self-compassion and hope in infertile women.	Quantitative, cross-sectional descriptive-correlational; SEM Level of evidence (4)	Inclusion: ≥ 3 years marriage; ≥ 1 year infertility; desire for children; age 20–40 Exclusion: infertility-related physical problems; psychiatric disorders; psychiatric medication Setting: infertility centres (Yazd, Fars, Kerman, Isfahan, Iran)	*N* = 346 infertile women Mean age: 28.56 ± 3.93 Treatment history: • First treatment: *n* = 51 • One failed attempt: *n* = 53 • 2–4 failed attempts: *n* = 131	SCS HS AAQ‑II	Self-compassion positively associated with acceptance (r = 0.69, *p* < 0.01) and hope (r = 0.29, *p* < 0.05) Acceptance positively associated with hope (r = 0.31, *p* < 0.05) Self-compassion significantly predicted acceptance and hope Acceptance mediated the relationship between self-compassion and hope (indirect r = 0.36, *p* < 0.05)
Raque-Bogdan and Hoffman (2015) USA	Examine differences in fertility-related stress by infertility type (primary or secondary) and role of self-compassion in predicting subjective well-being.	Quantitative, correlational cross-sectional Level of evidence (4)	Inclusion: women with primary or secondary infertility, not seeking treatment Setting: online survey via infertility support groups	*N* = 172 women Primary infertility *n* = 119; Secondary *n* = 53 Age: Primary: 31.81 ± 5.51 (range 21–51) Secondary: 33.76 ± 5.89 (range 21–47), significantly older (t = 2.27, *p* = 0.02) Trying to conceive: Primary 22.79 ± 20.78 months Secondary 38.28 ± 33.44 months	SCS FPI SWLS PANAS	Higher self-compassion associated with higher subjective well-being (large effect) Higher self-compassion associated with lower global infertility stress (large effect in primary infertility; medium effect in secondary infertility) Self-compassion associated with lower infertility-related stress across domains (large reductions in social and sexual concerns; moderate reductions in relationship concerns and need for parenthood) Self-compassion and infertility-related stress predicted well-being (R^2^ = 0.56, *p* < 0.001) Self-compassion mediated the relationship between infertility-related stress (need for parenthood, social concern) and well-being No significant group differences in self-compassion, well-being, or global infertility stress (*p* ≥ 0.61)
Galhardo et al. (2013b) Portugal	Examine gender differences in mediating roles of self-compassion and self-judgement between shame, dyadic adjustment and infertility-related stress.	Quantitative, cross-sectional; path analysis (SEM) Level of evidence (4)	Inclusion criteria: ≥18 yrs; infertility diagnosis Settings: public and private infertility clinics	*N* = 309 (women *n* = 162 primary infertility; men *n* = 147) Mean age: 34.51 ± 4.99 yrs Relationship duration: 6 ± 3.56 yrs Infertility duration: 2.96 ± 2.83 yrs Treatment: 72.5% treated; 27.5% pre-treatment	SCS OAS ESS DAS FPI	Self-compassion (women) fully mediated the relationship between internal shame and infertility-related stress (b = 0.11, 95% CI = 0.048–0.187) Self-compassion partially mediated the association between dyadic adjustment and stress (b = −0.05, 95% CI = −0.102 to −0.016) External shame had a direct effect on stress (effect = 0.30) Models explained variance in stress: 31% (women) and 18% (men) Women reported higher infertility-related stress and shame than men (*p* < 0.05) In men, self-judgement (not self-compassion) fully mediated the relationship between internal/external shame and stress (b = 0.08–0.12) Dyadic adjustment predicted lower stress in men (effect = −0.269)
Pinto-Gouveia et al. (2012) Portugal	Examine the role of self-compassion, psychological flexibility/acceptance and coping styles in depression and infertility-related self-efficacy.	Quantitative, cross-sectional Level of evidence (4)	Inclusion: couples with infertility diagnosis seeking treatment Setting: four public and three private infertility clinics	Infertile group: *n* = 200 (100 couples) Control group: *n* = 200 (100 couples) Mean age: • Infertile: 34.29 ± 5.04 • Control: 35.16 ± 4.37 Years of marriage: • Infertile: 6.14 ± 3.31 • Control: 8.88 ± 4.15	SCS BDI AAQ-II CSQ ISE	Infertile participants reported lower self-compassion than controls (t = 2.57, *p* = 0.010) Women reported lower self-compassion than men within the infertile group (t = 3.80, *p* = 0.000) Self-compassion negatively associated with depression in infertile participants Self-compassion positively associated with infertility self-efficacy Self-compassion predicted infertility self-efficacy in women (*β* = .48, *p* = 0.000) Psychological flexibility/acceptance (not self-compassion) predicted lower depression in men (*β* = −0.63, *p* = 0.000) and women (*β* = −0.45, *p* = 0.000)
Galhardo et al. (2011) Portugal	Examine the relationship between shame, self-judgement and psychological symptoms (depression and anxiety) in infertile couples, with comparisons to control and adoption groups.	Quantitative, cross-sectional Level of evidence (4)	Control group: general population Infertile group: recruited from public and private infertility clinics Adoption group: recruited from social services adoption offices Setting: public and private centres	Control: *n* = 200 (100 couples) Infertile: *n* = 200 (100 couples) Adoption: *n* = 80 (40 couples) Mean age: • Control: 35.16 ± 4.39 • Infertile: 34.24 ± 5.05 • Adoption: 36.64 ± 6.37 Years of marriage: • Control: 8.88 ± 4.15 • Infertile: 6.07 ± 3.31 • Adoption: 10.61 ± 5.43	SCS BDI STAI OAS ESS	Infertile couples reported higher depression, anxiety, shame and self-judgement than controls and adoption group Self-judgement (self-compassion component) was higher in infertile couples than controls (*p* = 0.000) In infertile couples, depression was positively associated with self-judgement, external shame and internal shame Self-judgement (b = 0.29, *p* = 0.000), external shame (b = 0.26, *p* = 0.001) and internal shame (b = 0.18, *p* = 0.022) predicted depression (R^2^ = 0.41) In the adoption group, self-judgement predicted anxiety (b = 0.44, *p* = 0.002; R^2^ = 0.31) Women reported higher depression, internal shame and self-judgement than men in the infertile group (*p* < 0.001)

List of abbreviated scales in the table: SCS – Self-Compassion Scale; DASS – Depression Anxiety Stress Scales; MRQ – Mindfulness Relationship Questionnaire; SCS-SF – Self-Compassion Scale–Short Form; PROMIS Depression – Patient-Reported Outcomes Measurement Information System Depression Scale; PROMIS Anxiety – Patient-Reported Outcomes Measurement Information System Anxiety Scale; PTGI – Posttraumatic Growth Inventory; AAQ-II – Acceptance and Action Questionnaire–II; IUS-SF – Intolerance of Uncertainty Scale–Short Form; mDES – modified Differential Emotions Scale; CSI-4 – Coping Strategies Inventory–4; FFMQ-SF – Five Facet Mindfulness Questionnaire–Short Form; ERQ – Emotion Regulation Questionnaire; Positivity Scale – Positivity Scale; CES-D-R – Centre for Epidemiologic Studies Depression Scale–Revised; K6 – Kessler Psychological Distress Scale (6-item version); RAS – Relationship Assessment Scale; HS – Hope Scale; FPI – Fertility Problem Inventory; SWLS – Satisfaction With Life Scale; PANAS – Positive and Negative Affect Schedule; OAS – Other As Shamer Scale; ESS – External Shame Scale; DAS – Dyadic Adjustment Scale; BDI – Beck Depression Inventory; CSQ – Coping Strategies Questionnaire; ISE – Infertility Self-Efficacy Scale; STAI – State–Trait Anxiety Inventory.

### Participants' characteristics

Across the intervention studies, a total of 591 women with infertility participated, with sample sizes ranging from 24 to 200 participants. All participants were female, with mean ages ranging from 31 to 35 years, and infertility duration ranging from 1 to 9 years, including both primary and secondary. While the cross-sectional studies included a total of 3005 participants, were enroled, predominantly women with two studies included male partners and general populations controls. Sample size ranged from 115 to 480, with mean ages between 28 and 35 years, with infertility duration of 3 to 4 years.

### Outcome(s) measures

The included studies used different versions of self-compassion scales (SCS). Ten studies used the SCS, including the 26 item version (Galhardo et al., 2011, 2013a, 2013b; Hajihasani & Ekhtiari Amiri, 2023; Pinto-Gouveia et al., 2012; Raque-Bogdan & Hoffman, 2015; Sotoudeh et al., 2022), the 12 item version (Njogu et al., 2023; Schuette et al., 2023) and a Chinese version (Li et al., 2016), while four studies delivered self-compassion interventions without reporting SCS (Afshani et al., 2019; Rezapour-Mirsaleh et al., 2020, 2024; Sahraian et al., 2024). Additional measures assessed depression, anxiety, infertility and QoL. Depression and anxiety were commonly measured using validated scales such as the Beck Depression Inventory scale (21 items) (Galhardo et al., 2011, 2013a; Pinto-Gouveia et al., 2012; Rezapour-Mirsaleh et al., 2020), and Zung Self-Rating Depression Scale (20 items (Njogu et al., 2023). Other constructs included Acceptance and Action Questionnaire II, and shame (Galhardo et al., 2011, 2013a, 2013b; Pinto-Gouveia et al., 2012; Sotoudeh et al., 2022). Infertility-related outcomes included the Self-Efficacy Scale (16 item) (Galhardo et al., 2013a; Njogu et al., 2023; Pinto-Gouveia et al., 2012) and fertility Problem Inventory scale (46 items) (Galhardo et al., 2013b; Raque-Bogdan & Hoffman, 2015). QoL was assessed using Fertility QoL Tool (24 items) (Li et al., 2016), QoL Questionnaire in Infertile Couples Questionnaire (72 items) (Rezapour-Mirsaleh et al., 2024, 2020) and marital relationship quality questionnaire (Hajihasani & Ekhtiari Amiri, 2023).

### Intervention studies (*N* = 7)

#### Intervention and mode of delivery

Seven intervention studies incorporated self-compassion education with varying approaches and durations. The included studies used various methods of delivery for the intervention, such as face-to-face, online, or mixed methods. The mode of delivery varied using lectures, giving feedback, workshop manual or group discussions or using videos, CD and internet-based platforms such as Zoom. Six studies provided the education intervention utilising face-to-face (Afshani et al., 2019; Galhardo et al., 2013a; Li et al., 2016, Rezapour-Mirsaleh et al., 2020, 2024; Sahraian et al., 2024), while only one study (Njogu et al., 2023) was conducted online.

The intervention duration ranged from six to ten sessions, with each session lasting between one and two and a half hours. Relaxation strategies were a common component of these interventions, often involving daily self-compassion exercises or homework exercises. The interventions were led by certified or trained specialists, such as clinical psychologists, nurses, or family counsellors (Galhardo et al., 2013a; Li et al., 2016; Njogu et al., 2023; Rezapour-Mirsaleh et al., 2020; Sahraian et al., 2024), except for two studies that did not report on who provided the education (Afshani et al., 2019; Rezapour-Mirsaleh et al., 2024). All studies reported the data collection time points of pre- and post- intervention. Only two studies reported details of a follow-up at 10 weeks after the intervention (Njogu et al., 2023; Sahraian et al., 2024) (Table 1 Characteristics of intervention studies).

#### Intervention effectiveness on women experiencing infertility

1. **Effect of self-compassion intervention on depression, anxiety and shame**

Three studies revealed that self-compassion interventions significantly decreased depressive symptoms in women experiencing infertility (Galhardo et al., 2013a; Njogu et al., 2023; Sahraian et al., 2024). Significant improvements were observed immediately after the self-compassion intervention (*p* < 0.001) and were maintained at follow-up (Njogu et al., 2023). Galhardo et al. (2013a) also reported a significant decrease in depression scores within the intervention group, from pre-intervention (T1: M = 11.02) to post-intervention (T2: M = 6.18) (Galhardo et al., 2013a).

Four studies reported a positive effect of self-compassion interventions on reducing anxiety in women experiencing infertility (Galhardo et al., 2013a; Njogu et al., 2023; Rezapour-Mirsaleh et al., 2020, 2024). Women in the self-compassion training using video (SCV) and self-compassion training using digital stories (SC-DS) groups experienced a significant reduction in anxiety from T1 (pre-intervention) to T2 (post-intervention) and from T1 to T3 (follow-up) (*p* < 0.001) (Njogu et al., 2023). Similarly, significant decreases in anxiety were reported within the intervention group (F = 68.54, *p* < .001, and Partial *η* 2 = .77) (Galhardo et al., 2013a; Rezapour-Mirsaleh et al., 2020). Another study found that the religious self-compassion group showed greater reductions in anxiety compared to the non-religious group (Rezapour-Mirsaleh et al., 2024).

Galhardo et al. examined the effectiveness of self-compassion intervention on several psychological factors in women experiencing infertility, including entrapment, shame, defeat and psychological inflexibility (Galhardo et al., 2013a). The study reported a significant decrease in external entrapment (*p* = .009) and internal entrapment (*p* = .021) from T1 to T2 in the intervention group (Galhardo et al., 2013a). Similarly, there was a significant reduction in internal shame (*p* = .001) including character, behaviour and body, as well as a decrease in external shame (*p* = .011). In addition, the intervention group showed a significant decrease (*p* < .001) in experiential avoidance and feeling of defeat (*p* = .007) from T1 to T2 (Galhardo et al., 2013a).

2. **Effect of self-compassion intervention on psychological well-being**

One study reported a significant positive effect of a self-compassion intervention on psychological well-being among women experiencing infertility (Afshani et al., 2019). In the intervention group, psychological well-being scores increased from pre-test (M = 316.94) to post-test (M = 349.88), whereas the control group showed minimal change from pre-test (M = 317.38) to post-test (M = 320.88). This difference between groups was statistically significant (*p* = 0.01).

3. **Effect of self-compassion intervention on QoL and self-efficacy**

Self-compassion interventions were associated with improvements in QoL and self-efficacy. Three studies examined the effect of self-compassion interventions on QoL among women experiencing infertility (Rezapour-Mirsaleh et al., 2024; Li et al., 2016; Rezapour-Mirsaleh et al., 2020). Rezapour-Mirsaleh et al. (2020) reported a significant improvement in QoL in the intervention group following the intervention (F = 57.22, *p* < .001, and Partial *η* 2 = .74) (Rezapour-Mirsaleh et al., 2020). Similarly, Li et al. (2016) found that QoL scores significantly increased in the intervention group from pre-test (M = 59.04) to post-test (M = 71.72; *p* < .001) (Li et al., 2016). More recently, Rezapour-Mirsaleh et al. (2024) reported that the religious self-compassion group demonstrated greater improvements in overall QoL compared to the non-religious group (Rezapour-Mirsaleh et al., 2024).

Two studies examined the effect of self-compassion intervention on self-efficacy in women experiencing infertility (Galhardo et al., 2013a; Njogu et al., 2023). One study reported significant improvements in self-efficacy from pre-intervention (T1) to post-intervention (T2) and from T1 to follow-up (T3) (*p* < 0.001) (Njogu et al., 2023). Similarly, Galhardo et al. (2013a) reported a significant increase in self-efficacy following the intervention from T1 (M = 81.87) to T2 (M = 24.45) (*p* < .001).

4. **Effect of self-compassion intervention on emotion regulation and coping strategies**

Two studies reported significant positive effects of self-compassion interventions on emotional regulation and coping strategies for women experiencing infertility (Afshani et al., 2019; Li et al., 2016). Li et al. (2016) reported a significant reduction in emotion regulation difficulties in the intervention group from pre-intervention (M = 92.00) to post-intervention (M = 78.76; *P* < .001). Similarly, Afshani et al. (2019) found that self-compassion training significantly reduced negative emotions among participants.

Regarding infertility-related coping strategies, Li et al. (2016) reported significant positive effects of a self-compassion intervention on women experiencing infertility. Women in the Mindfulness-Based Intervention group for IVF demonstrated significant reductions in active-avoidance coping (*p* = 0.001) and passive-avoidance coping (*p* = 0.010), as well as a significant increase in meaning-based coping (*p* < 0.001). However, no significant change was observed in active-confronting coping strategies such as expressing emotions (*p* = 0.959).

5. **Effect of self-compassion intervention on self-compassion and self-judgement**

Three intervention studies examined self-compassion as an outcome measure (Galhardo et al., 2013a; Li et al., 2016; Njogu et al., 2023). Njogu et al. (2023) reported significant increases in self-compassion from pre-intervention (T1) to post-intervention (T2) and from T1 to follow-up (T3) (*p* < 0.001, d > 0.8). However, no statistically significant differences were observed between the self-compassion videos (SCV) group and the self-compassion training using digital stories (SC-DS) (*p* > 0.05) (Njogu et al., 2023). Similarly, Li et al. (2016) reported that self-compassion scores in the intervention group increased significantly from pre-intervention (M = 3.06) to post-intervention (M = 3.60; *p* < 0.001). Galhardo et al. (2013a) reported a significant reduction in self-judgement, a sub-scale of self-compassion, from pre-intervention (M = 38.49) to post-intervention (M = 34.38; *p* < 0.001) in the intervention group compared to the control group. However, no significant differences were observed in overall self-compassion scores (Galhardo et al., 2013a).

6. **Effect of self-compassion intervention on pregnancy rates**

Only two studies reported the effect of self-compassion intervention on pregnancy rates among women with infertility (Li et al., 2016; Njogu et al., 2023). Li et al. (2016) reported a significant increase in viable pregnancies at six months post-intervention (*p* = 0.04), with a rate of 44.83% in the intervention group compared to 26% in the control group (Li et al., 2016). In contrast, Njogu et al. (2023) reported no significant impact of their self-compassion intervention on pregnancy rates (Njogu et al., 2023).

### Cross-sectional studies (*N* = 9)

Nine included studies were cross-sectional and did not involve self-compassion interventions. However, these studies examined the association between self-compassion and fertility-related stress, anxiety, depression, well-being, self-efficacy, hope and acceptance of infertility-related problems (Table 2). The findings from the cross-sectional studies are presented below:

#### Relationship between self-compassion and fertility-related stress and anxiety

Six studies explored the relationship between self-compassion and infertility-related stress (Galhardo et al., 2011, 2013b; Hajihasani & Ekhtiari Amiri, 2023; Hoyle et al., 2022; Raque-Bogdan & Hoffman, 2015; Sadiq et al., 2022). Higher levels of self-compassion were associated with lower infertility-related stress, particularly among women with primary infertility (Raque-Bogdan & Hoffman, 2015). Self-compassion was also negatively associated with social, sexual and relationship concerns, as well as the perceived need for parenthood, although the association was weaker for rejection of a child-free lifestyle. Similarly, higher self-compassion was associated with lower levels of external and internal shame and infertility-related stress (Galhardo et al., 2013b). Among infertile couples pursuing adoption, anxiety was moderately and positively associated with self-judgement, including self-criticism, isolation and over-identification (Galhardo et al., 2011).

#### Relationship between self-compassion, self-judgement and depression

Six studies explored the relationship between self-compassion and depressive symptoms in infertile couples (Galhardo et al., 2011; Hajihasani & Ekhtiari Amiri, 2023; Hoyle et al., 2022; Pinto-Gouveia et al., 2012; Sadiq et al., 2022; Schuette et al., 2023). These studies reported that self-compassion was a significant negative predictor of depression, anxiety and stress among women with primary infertility. In addition, depressive symptoms were positively and moderately associated with self-judgement in infertile couples (Galhardo et al., 2011).

#### Relationship between self-compassion and well-being, self-efficacy, hope and acceptance

Self-compassion demonstrated a large effect on subjective well-being among women with primary and secondary infertility (Raque-Bogdan & Hoffman, 2015). In addition, self-compassion mediated the relationship between the need for parenthood, subjective well-being and social concerns. Self-efficacy was also positively associated with self-compassion, with regression analysis showing that self-compassion was the only significant predictor of self-efficacy (*β* = .48; *p* = .000) (Pinto-Gouveia et al., 2012). The model explained 46% of the variance in self-efficacy, indicating a strong association between self-compassion and confidence in managing infertility-related challenges.

Sotoudeh et al. (2022) examined the relationship between self-compassion, hope and acceptance of infertility-related problems among women experiencing infertility (Sotoudeh et al., 2022). The study found that higher self-compassion scores (M = 72.12, SD = 15.942) were significantly associated with greater hope (r = 0.29, *p* < 0.05), and higher acceptance of infertility-related problems (r = 0.69, *p* < 0.01).

## Discussion

This systematic review synthesised evidence on the influence of self-compassion in supporting the mental health and well-being of women experiencing infertility. The evidence indicates a consistent association between higher self-compassion and improved mental health and well-being outcomes. Self-compassion appears to positively influence on women's levels of anxiety, depression, shame and infertility-related stress, while also improving coping, self-efficacy, emotion regulation and quality of life. Utilising self-compassion strategies and developing skills, women experiencing infertility may gain a protective resource for mental health and wellbeing.

Self-compassion may exert its beneficial effects through several interconnected mental health state mechanisms. Evidence from the included studies indicates that self-compassion is associated with reductions in emotional distress alongside improvements in coping and emotion regulation (Afshani et al., 2019; Li et al., 2016). By encouraging individuals to respond to difficult experiences with self-kindness rather than self-criticism, self-compassion may reduce maladaptive cognitive processes such as rumination and self-blame, which are commonly reported among women experiencing infertility. In addition, the mindfulness component of self-compassion may support greater emotional awareness and regulation by enabling individuals to acknowledge distress without becoming overwhelmed by it, which may explain the observed reductions in anxiety, depression and shame across intervention studies. The emphasis on common humanity may also play an important role by helping women reframe infertility as a shared human experience rather than a personal failure, thereby reducing feelings of isolation and stigma. These processes suggest that self-compassion enhances psychological flexibility and coping capacity, supporting improved mental health and well-being in this target population.

This review contributes further evidence to current literature that has reported positive mental health and well-being outcomes associated with self-compassion across diverse populations across diverse populations (Ahmed & Raj, 2023; Othman et al., 2022; Sinclair et al., 2021; Steen et al., 2021). For example, self-compassion has been shown to reduce guilt-related trauma and moral distress among veterans (Steen et al., 2021), alleviate anxiety and stress in nurses and midwives while buffering against compassion fatigue and burnout (Steen et al., 2021), support parents of children with additional needs in managing daily challenges and emotional distress (Ahmed & Raj, 2023; Othman et al., 2022), parents to manage grief after experiencing a perinatal loss (Roberts, 2025) and helps older adults cope with chronic illness, anger and increase resilience (Othman et al., 2025). Within the context of infertility, self-compassion may therefore act both as an emotion regulation strategy and a source of resilience, fostering a more understanding and non-judgemental relationship with oneself while alleviating emotional distress and feelings of shame associated with infertility and its treatments.

The studies included in this review, published over the past decade, reflect a growing research interest in the influence of self-compassion on infertility. However, the evidence base remains relatively limited in scope and depth. Most studies were conducted in clinical settings, such as fertility centres or outpatient clinics, which may not fully capture the broader experiences of women managing infertility in community or home environments. Expanding research into more diverse contexts, including community-based and home-based settings, may provide a more comprehensive understanding of how self-compassion is applied and sustained in everyday life. In addition, incorporating partners into future interventions may offer valuable insight into the relational dimensions of infertility and coping.

A notable gap in the literature is the limited inclusion of men and gender-diverse individuals. The small number of studies examining gender differences suggests that psychological responses to infertility, and the role of self-compassion, may differ across groups. For instance, women have been reported to experience higher levels of infertility-related stress, shame and self-judgement, alongside lower levels of self-compassion compared to men (Galhardo et al., 2013b). In contrast, findings indicate that self-compassion may buffer the impact of internal shame among women, whereas negative self-relational processes appear to function more strongly as risk factors in men, intensifying the relationship between shame and infertility-related stress (Pinto-Gouveia et al., 2012). These findings highlight the need for more inclusive research that considers gender and relational dynamics.

Only one study in this review reported the delivery of a self-compassion intervention via an online platform, highlighting an educational access gap (Njogu et al., 2023). This is notable given the increasing reliance on digital health approaches to improve accessibility and engagement in psychological interventions (Beshai et al., 2020; Li et al., 2021). The limited use of online modalities within this target population suggests missed opportunities to extend support to women who may face barriers to accessing in-person services, particularly during prolonged or demanding fertility treatment processes.

The heterogeneity of intervention designs, delivery modes and outcome measures across studies makes it difficult to determine which components are most effective. However, the review suggests that interventions incorporating elements such as guided meditation, relaxation techniques, structured self-compassion exercises and opportunities for shared experiences may contribute to positive psychological outcomes (Galhardo et al., 2013a; Li et al., 2016). These components are likely to work synergistically to support emotional regulation and reduce distress.

The available evidence indicates that self-compassion interventions have the potential to improve emotional well-being and coping among women undergoing infertility treatment. Fostering a less self-judgemental and more accepting stance towards one's experiences may enhance psychological adjustment. Interventions that integrate multiple components and are tailored to individual needs appear particularly promising, although further research is required to identify optimal formats and delivery approaches.

## Strengths and limitations of the review

This review was conducted in accordance with a predefined protocol and guided by Joanna Briggs Institute (JBI) methodology, incorporating systematic search strategies and critical appraisal tools to enhance methodological rigour. The findings consistently indicate positive associations between self-compassion and mental health outcomes among women experiencing infertility.

However, several limitations should be considered. Only studies published in English were included, which may have resulted in the exclusion of relevant research from non-English sources. In addition, the included studies were conducted across a limited number of countries, restricting the generalisability of findings to broader culturally and linguistically diverse populations. Substantial heterogeneity in study design, intervention characteristics and outcome measures precluded the conduct of a meta-analysis and limited direct comparability across studies. Furthermore, the findings are based on relatively small samples and varied methodological approaches, which may influence the robustness of the conclusions. Potential publication bias should also be considered, as studies reporting positive psychological outcomes may be more likely to be published. This may result in an overrepresentation of favourable findings and a possible overestimation of the effectiveness of self-compassion interventions.

## Conclusions

This review synthesised evidence from 16 studies examining the influence of self-compassion in women experiencing infertility, indicating consistent associations with reduced psychological distress and improved coping-related outcomes. While intervention studies suggest beneficial effects, the evidence is limited by small sample sizes, heterogeneous designs and short follow-up periods, restricting confidence in effect stability and broader applicability.

The predominance of studies conducted in clinical settings and a limited number of countries further constrains generalisability across diverse populations and contexts. Future research should prioritise rigorously designed randomised controlled trials with standardised outcome measures, longer-term follow-up and more diverse populations, including under-represented groups. Self-compassion represents a promising but still evolving area of intervention, requiring further methodological refinement before it can be confidently integrated into routine infertility care.

## Supplementary Material

Supplementary Material PRISMA_2020_checklist.docx

## Data Availability

There is no data associated with this research.

## Impact statement

This systematic review demonstrates that self-compassion intervention can improve psychological well-being and reduce emotional distress among women experiencing infertility. Integrating self-compassion education into fertility care may strengthen resilience and provide a valuable, evidence-based approach to supporting mental health in this population.

## PROSPERO registration

This systematic review has been registered in the International Prospective Register of Systematic Reviews (PROSPERO) (ID: CRD42022330158).

## Relevance to clinical practice

These findings have important implications for clinical practice. Integrating self-compassion-based approaches into fertility services, including counselling and psychosocial support programmes, may offer a valuable strategy for improving psychological well-being. Structured interventions incorporating mindfulness practices, guided reflection and peer support could be embedded within routine care to support women throughout the infertility and treatment process. Expanding access through digital platforms may further enhance reach and accessibility.

## Abbreviations

IVF: In Vitro Fertilisation
JBI: Joanna Briggs Institute
MSC: Mindful Self-Compassion
QOL: Quality of Life
PRISMA: Preferred Reporting Items for Systematic Review and Meta-Analysis
SCS: Self-Compassion Scale
SC-DS: Self-Compassion training using Digital Stories
SCV: Self-Compassion training using Videos

## Appendices

### Appendix A: ovid MEDLINE search strategy

**Table:**

#	Query	Results from 18/11/24
1	exp Infertility, Female/or exp Infertility/	76,554
2	exp Sperm Injections, Intracytoplasmic/	8,302
3	exp Reproductive Techniques, Assisted/or exp Fertilisation in Vitro/or exp Embryo Transfer/	83,768
4	exp Insemination, Artificial/	12,771
5	exp Oocyte Donation/	2,909
6	(infertile or infertility or in vitro or infertil* or subfertil* or sterility or reproductive sterility or infertile woman or infertile women or infertile female or female infertility or ICSI or intracytoplasmic sperm injection or artificial insemination or vitro fertilisation or vitro fertilisation or IVF or assisted reproduct* or intrauterine insemination).ti,ab,kf.	1,639,194
7	1 or 2 or 3 or 4 or 5 or 6	1,683,037
8	exp Self-Compassion/	380
9	(self-compassion or self compassion or self-kindness or self kindness or self-regard or self regard or self-worth or self worth or self-appreciation or self appreciation or self-care or self care or self-acceptance or self acceptance or self-forgiveness or self-forgiveness).ti,ab,kf.	34,166
10	8 or 9	34,167
11	7 and 10	84

### Appendix B: critical appraisal checklist tables

**Table B1.** JBI critical appraisal checklist for randomised controlled trials and Quasi-experimental studies.

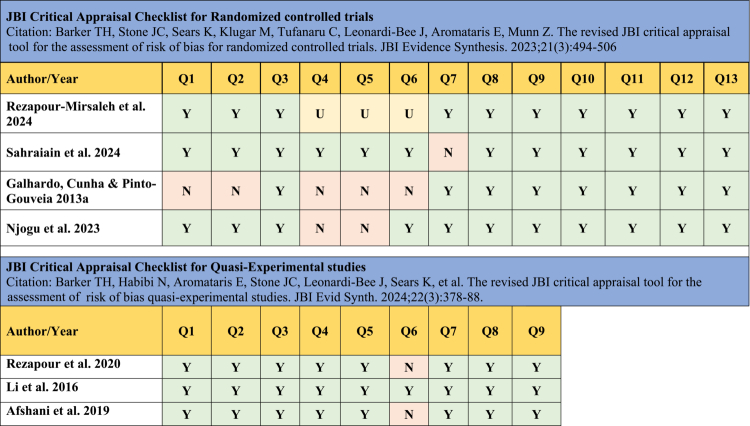

Y = YES, N = NO, U = UNCLEAR, NA = Not applicable.

**Table B2.** JBI Critical Appraisal Checklist for Cross-sectional studies (n=9).

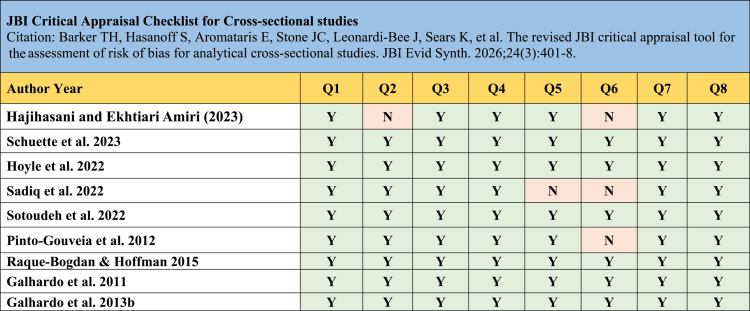

Y = YES, N = NO, U = UNCLEAR, NA = Not applicable.
